# A Simple Sensor System for Onsite Monitoring of O_2_ in Vacuum-Packed Meats during the Shelf Life

**DOI:** 10.3390/s21134256

**Published:** 2021-06-22

**Authors:** Elisa Santovito, Sophia Elisseeva, Malco C. Cruz-Romero, Geraldine Duffy, Joseph P. Kerry, Dmitri B. Papkovsky

**Affiliations:** 1 School of Biochemistry and Cell Biology, University College Cork, T12 YT20 Cork, Ireland; Elisa.santovito@ucc.ie (E.S.); sophia.elisseeva@ucc.ie (S.E.); 2 Food Packaging Group, School of Food and Nutritional Sciences, University College Cork, T12 YT20 Cork, Ireland; m.cruz@ucc.ie (M.C.C.-R.); joe.kerry@ucc.ie (J.P.K.); 3 Teagasc Food Research Centre, Food Safety Department, Ashtown, D15 KN3K Dublin, Ireland; geraldine.duffy@teagasc.ie

**Keywords:** phosphorescence-based oxygen sensor, nondestructive oxygen measurement, food packaging, residual oxygen levels, vacuum-packed meat

## Abstract

Vacuum packaging (VP) is used to reduce exposure of retail meat samples to ambient oxygen (O_2_) and preserve their quality. A simple sensor system produced from commercial components is described, which allows for non-destructive monitoring of the O_2_ concentration in VP raw meat samples. Disposable O_2_ sensor inserts were produced by spotting small aliquots of the cocktail of the Pt–benzoporphyrin dye and polystyrene in ethyl acetate onto pieces of a PVDF membrane and allowing them to air-dry. These sensor dots were placed on top of the beef cuts and vacuum-packed. A handheld reader, FirestinGO2, was used to read nondestructively the sensor phase shift signals (dphi°) and relate them to the O_2_ levels in packs (kPa or %). The system was validated under industrial settings at a meat processing plant to monitor O_2_ in VP meat over nine weeks of shelf life storage. The dphi° readings from individual batch-calibrated sensors were converted into the O_2_ concentration by applying the following calibration equation: O_2_ (%) = 0.034 * dphi°^2^ − 3.413 * dphi° + 85.02. In the VP meat samples, the O_2_ levels were seen to range between 0.12% and 0.27%, with the sensor dphi signals ranging from 44.03° to 56.02°. The DIY sensor system demonstrated ease of use on-site, fast measurement time, high sample throughput, low cost and flexibility.

## 1. Introduction

Rising global production and consumption of meat over the past decade has introduced new challenges in the meat supply chain [1]. Like all perishable foods of animal origin, fresh meat is susceptible to microbial contamination and spoilage and related processes like lipid oxidation, sensory and quality degradation, which occur at different stages of the production chain including preparation, storage and distribution [2]. Therefore, quality control and safety assurance of foods of animal origin are necessary for reducing the losses, mitigating health risks and ensuring consumers’ safety. Consequently, new packaging and monitoring technologies are actively being deployed to support shelf life extension of products, improve and maintain their quality and safety [3,4,5,6,7]. 

By reducing the exposure of meat to ambient oxygen, vacuum packaging (VP) helps to preserve the organoleptic properties of the product and reduce microbial growth and other spoilage-related degradative processes [8]. VP is therefore a common packaging method in the meat industry, especially for the transport and sale of fresh meat products, preservation of quality and extension of their shelf life up to 55 days. [9]. VP can extend the storage period of raw meat samples by 40–55 days [10,11,12]. In addition, VP helps preserve colour, which is an important visual quality of raw meat used by consumers at the point of sale [13], and oxygen-dependent meat browning leads to significant rejection of the product by consumers [14,15]. 

At the same time, VP does not mean full removal of all the residual O_2_ from individual packs as it can be trapped within the food and the packaging material or permeate from air atmosphere into the packaging material during product storage depending on the barrier properties of the packaging material [16]. Therefore, smart VP systems which can nondestructively determine and monitor the residual O_2_ levels in packaged products at different stages of their shelf life are of high importance for the food production chain, especially for raw meat products. Monitoring of O_2_ inside the package can also be used to predict the growth of microbiota in packs [17,18,19]. 

Smart packaging with nondestructive O_2_ sensors can reduce the risk of recalls, with the consequent benefits for the company’s reputation [17]. However, for the O_2_ sensors to be commercially viable and adoptable by the food industry, their benefits must outweigh the additional costs associated with this technology [20]. Some O_2_ sensors are commercially available or can be manufactured on a large scale using relatively inexpensive materials and equipment [8]. Robust, long-term and periodic monitoring of the residual O_2_ levels in packs with disposable O_2_ sensors has been demonstrated in various food and nonfood applications [17,19,21,22,23]. Optical O_2_ sensing systems have previously been applied using commercial O_2_ sensors to monitor the evolution of meat spoilage in VP samples of raw beef and to analyse the efficiency of different vacuum packaging machines [24]. At the same time, very few studies have been performed with O_2_ sensor systems applied on-site to monitor the quality of the VP meat produced under large-scale industrial settings. 

We present a simply constructed O_2_ sensor system designed for monitoring the residual O_2_ in VP raw beef cuts and quality assessment of these products during their shelf life by means of disposable, low-cost, in-house-made sensors. The application of the sensor system was performed directly at the meat packaging line, where fresh beef cuts were produced and vacuum-packed together with disposable O_2_ sensors placed in every pack. Then, the samples were transported to a research meat processing plant and the following quality parameters were analysed over nine weeks of storage: (i) residual O_2_ measured nondestructively in each pack with in-house-made disposable O_2_ sensors interrogated with a handheld FiresingGO2 reader, (ii) microbial load (total viable count, Enterobacteriaceae, lactic acid bacteria and psychrotrophic bacteria) determined by the standard plate counting method, (iii) pH values of the meat samples and (iv) colour changes of meat assessed using the colorimetric analysis. 

## 2. Materials and Methods

### 2.1. Materials

The maximum recovery diluent (MRD), plate count agar (PCA), nutrient broth (NB), de Man, Rogosa and Sharpe agar, MRSA and PVDF membranes were from Sigma-Aldrich (Dublin, Ireland). The Enterobacteriaceae RAPID’Chromogenic Medium was from Bio-Rad Laboratories (United Kingdom). All the other chemicals and solvents were of analytical grade; the solutions were prepared using Milli-Q water. 

### 2.2. O_2_ Sensor Preparation and Calibration

**Figure 1:**
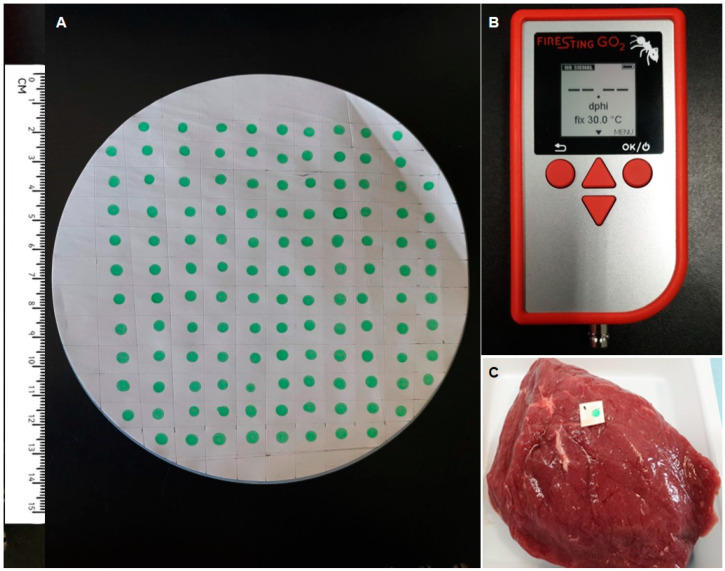
The low-cost O_2_ sensor system and measurement setup. (**A**) The array of sensor dots on the PVDF membrane. (**B**) The FirestingGo2 handheld reader used to measure sensor signals (dphi°) and convert them into the O_2_ concentration (kPa or %). (**C**) A disposable sensor dot placed on a meat cut prior to vacuum packaging.

One milligram of the Pt–benzoporphyrin dye (Frontier Scientific) was dissolved in 2.0 mL 5% (*w*/*w*) solution of polystyrene in ethyl acetate (Sigma-Aldrich). This cocktail was spotted in 3.0-µL aliquots onto sheets of 0.45-µm PVDF membrane (Millipore) using a Distriman^TM^ repetitive pipette (Gilson) and allowed to air-dry for 30 min under a laminar flow hood to ensure sterility. After this, the sensors were incubated for 24 h in a dry oven at 70 °C. Thus, arrays of O_2_ sensor dots (approx. 5 mm in diameter) were produced. The quality of sensor dots was assessed by measuring their intensity (mV) and phase shift (dphi°) signals in the air atmosphere using a FireStingGO2 reader (PyroScience, Germany). Batches of 50–100 disposable sensor dots thus produced (Figure 1A) were cut into individual pieces (approx. 10 × 10 mm each, Figure 1C) and stored in a dark place at room temperature until further use (for up to 12 months). 

The O_2_ calibration was carried out by placing one sensor dot in a 10-mL testing vial on a water bath equilibrated at 4 °C and purging the vial sequentially with the standard O_2_/N_2_ gas mixtures produced using an LNI precision gas mixer/tonometer (Switzerland) and humidified by bubbling them through distilled water, gradually changing the O_2_ content from 21.0 kPa (21%) to 0.00 kPa (0%). After the O_2_ equilibration of the vial (stable dphi° readings), steady-state dphi° signals were recorded with FireStingGO2, plotted as the function of the O_2_ concentration and fitted with a mathematical function. Two sensors from each batch were calibrated, and an average calibration for one batch was used to work out the analytical equation relating the O_2_ concentration in VP samples and the sensor dphi° signals. This is described in more detail in Section 3.2.

### 2.3. Preparation and Testing of Vacuum-Packed Meat Samples

Processing and packaging of fresh beef samples (boned out 48 h after slaughtering, stored at 0 °C) was carried out directly at the processing line of a large Irish export meat processing factory using its standard packaging equipment, packaging materials and process settings. Following the routine procedures for meat packaging at the production plant, boneless beef cuts (called chuck1, chuck2, knuckle, silverside and topside) were divided into smaller pieces of approximately 200 g each. One O_2_ dot sensor was applied on the surface of each piece of meat (Figure 1C) placed in a vacuum-packaging pouch, and then the pouches were sealed on a VP machine according to the established procedure. The VP fresh meat samples were transported in polystyrene boxes containing ice to the research meat processing plant at University College Cork where they were stored in a dark cold room at 1 ± 0.5 °C. All the packaged samples were analysed periodically for the residual O_2_ immediately after VP (day 0) and then weekly over a period of nine weeks. At each timepoint, O_2_ was measured nondestructively in all the VP samples; after this, several VP samples (one from each group) were opened and analysed for the other quality attributes (i.e., by destructive sampling).

### 2.4. Microbiological Analysis

For microbiological analyses of the VP meat samples, a 10-g sample was weighted, transferred into a stomacher bag containing 90 mL Maximum Recovery Diluent (Sigma-Aldrich, Dublin, Ireland), homogenized on Stomacher 400 (Stomacher Lab System, United Kingdom) for 2.5 min and plated on agar media in several 1:10 serial dilutions. Parallel to this, the MRS agar was used for colony counts of lactic acid bacteria (LAB) (ISO 15214, 1998), PCA was used for total mesophilic aerobic bacteria (TMAB), and the results were presented in log CFU (colony-forming units)/g values.

### 2.5. Meat pH Measurement

The pH of the VP meat cuts was measured with a digital pH-meter (Mettler-Toledo, Switzerland) by inserting its glass probe directly into the meat. Each pH value represents an average of three measurements per sample.

### 2.6. Measurement of the Meat Colour 

The colour of the VP meat (CIELAB colour space) was measured on a Chromameter CR-400 system (Konica Minolta Camera Co., Osaka, Japan), taking nine measurements for each sample or condition. Before the measurement, the instrument was calibrated according to the manufacturer’s instructions [25]. CIELAB colour space data with the L*, a* and b* values (lightness, redness and yellowness, respectively) were downloaded from the instrument and analysed. 

### 2.7. Data Analysis

The calibration equations relating the dphi° values measured from individual sensors/meat samples to the O_2_ concentration were obtained by fitting the calibration datapoints with the polynomial function. Data analysis and statistics were performed using Minitab 19 (Minitab Ltd., Coventry, UK). 

## 3. Results

### 3.1. Assembling a Low-Cost O_2_ Sensor System for Food Packaging Applications

The modular system for non-destructive sensing of the residual O_2_ levels in packaged food products was constructed on the basis of commercially available components, namely, (i) the phosphorescent O_2_-sensitive dye, Pt–benzoporphyrin (PtBP, Frontier Scientific), (ii) the sensor substrate/support material, a PVDF filter membrane (Millipore-Sigma), (iii) the sensor coatings, PtBP–polystyrene (Sigma) composite material dissolved in ethyl acetate and applied as discrete spots on the PVDF substrate (see Methods and [23]) and (iv) the FireStingGO2 sensor reader (Pyrosciences, www.pyroscience.com/en/products/all-meters/fsgo2, accessed 21 June 2021). The system setup is shown in Figure 1.

The sensor dye PtBP features high brightness and photostability, longwave excitation and emission bands in the red and near-infrared spectral range, near-optimal sensitivity to O_2_ in polymers such as polyolefines, and is affordable in price. For these reasons, PtBP is utilised by several vendors including PreSens, Mocon and Pyroscience [26]. However, commercial O_2_ sensors are expensive (€5–50 apiece), which prevents their use on the disposable basis and scale required by food packaging applications (dozens and hundreds of samples per trial). 

The same vendors also offer optical readers for PtBP-based sensors, which differ in their operational principles (phase or time domain), format (micro/macro sensors), flexibility (detachable/nondetachable/contactless) and other capabilities (temperature compensation, recalibration, etc.). Their general operational principles can be found in [26] or on the vendor’s website. The instrument price also varies from €1.5k to €15k, and they are usually marketed and bundled with the vendor’s own sensors. For our system, we selected the FireStingGO2 reader from Pyroscience (www.pyroscience.com/en/products/all-meters/fsgo2, accessed 21 June 2021) which features excellent optical performance, compact size, autonomous operation, open architecture and software along with an affordable price (€4.5k). The most affordable and more basic reader Piccolo2 (USB dongle, Pyroscience), which costs ~€1.5k, was also used in this study and found to provide similar results as FireStingGO2. 

Altogether, the above components gave us a low-cost DIY system alternative to the commercial O_2_ sensor systems [8] which can be used to assess the quality of VP meat cuts produced in small research labs and at large-scale industrial meat processing facilities. All the materials used to manufacture the DIY sensors did not require any modifications and were used as the manufacturer provided them.

### 3.2. Preparation and Characterization of Disposable O_2_ Sensor Dots 

Commercial O_2_ sensors normally function efficiently in the 0–21 kPa (0–21%) O_2_ range and have detection limits of 0.01–0.1 kPa [26], so their characteristics are appropriate for the standard meat packaging applications [6,23]. We produced in our lab multiple batches of disposable sensor dots (90–100 sensors in each batch) by spotting 3-µL aliquots of the cocktail containing sensor ingredients in ethyl acetate onto sheets of a PVDF filter membrane (Figure 1A). Prior to their use in VP meat samples, the uniformity and performance of these sensor dots was assessed with a FireStingGO2 reader (Figure 1B). The phase (dphi°) and intensity (mV) signals were measured in the air atmosphere (20.86% O2) at room temperature (22 °C) under the instrument’s default settings: LED intensity: 100%, amplification: 400×, frequency: 4 kHz. Three different batches of sensor dots, each batch consisting of 95 sensors, were analysed, measuring each sensor dot three times with ~3 s intervals between the readings. The measured data were downloaded from FireStingGO2 to a PC and analysed using the Minitab 19 software. 

**Figure 2:**
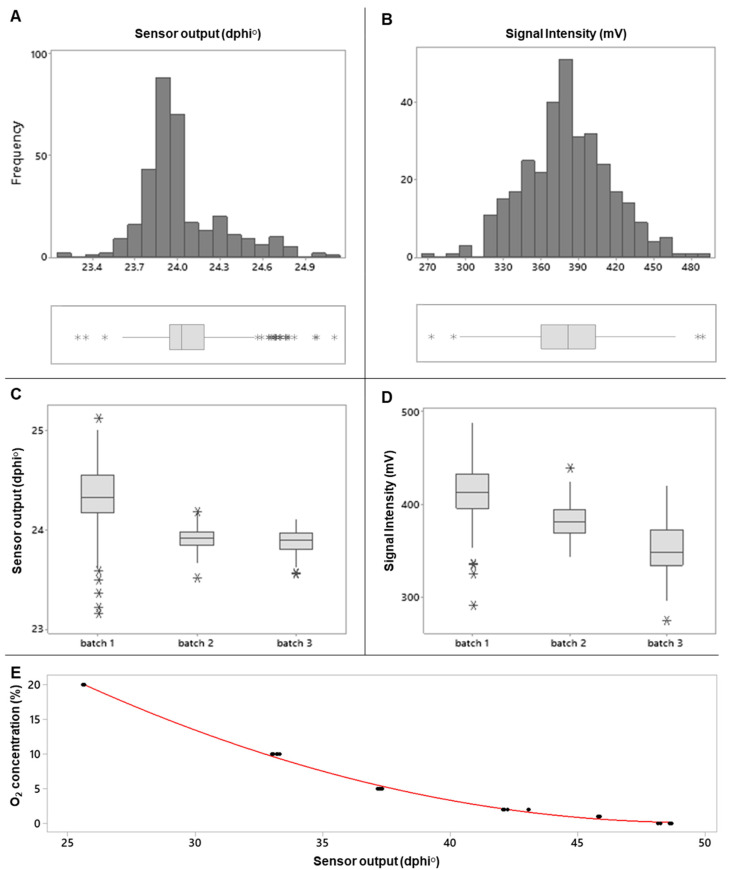
Initial quality check and O_2_ calibration for the disposable sensor dots. Quality check on sensor dots prior to use. The box plots show the distribution of the measured values. Histograms of the data (n = 137) show the distribution of the measured sensor dphi° (**A**) and intensity (mV) signals (**B**), and their corresponding box plots. Box plots (**C**,**D**) show the dphi° and mV values for the three batches (95 dots each) produced on three different days. The boxes show the interquartile data ranges (n = 100). The horizontal line in each of the boxes represents the median value. The whiskers represent the ranges for the bottom 25% and the top 25% of the data values, and 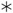 represent outliers. (**E**) The O_2_ calibration curve for sensor dots; the red line indicates the polynomial fitting curve.

The results of the quality check analysis of one batch of sensors are shown in Figure 2A,B. The mean sensor dphi° signal was 24.02 ± 0.29°, with the minimum value of 23.15° and the maximum value of 25.13°. The median value was 23.95° in the 95% confidence interval of 23.93–23.94°. The mean sensor intensity signal was 381.46 ± 34.40 mV, with the minimum value of 273.70 mV, the maximum value of 487.40 mV and the median value of 381.20 mV in the 95% interval confidence of 376.57 mV–383.96 mV. The ANOVA analysis for the three different batches of sensors indicated that there were no significant differences between the batches (Figure 2C,D). Thus, in-house-made O_2_ sensor dots can be produced in bulk and the manufcatured sensors provide consistent dphi° (and intensity) readings, which make them suitable for use in VP meat applications. Unlike the dphi° signals, sensor intensity signals in VP meat can be strongly influenced by the sample and measurement geometry, food color, etc. [26] so they cannot be used for accurate quantification of O_2_ in food packs. 

Potentially, reproducibility of individual sensors in the final packaging application can be further improved by excluding those sensors which give either very high or very low dphi° readings in the QC test (i.e., on both tails in Figure 2A). However, in this study, the exclusion of sensors with very high or very low dphi° readings was not carried out, and all the sensors from each batch were used. 

Next, we performed the O_2_ calibration for the combined batch of sensors (Figure 2E). For that, we randomly chose one sensor dot, equilibrated it at 4 °C, purged it with the standard O_2_/N_2_ gas mixtures with known O_2_ content (from 21.0 kPa (21%) to zero kPa). At each O_2_ concentration, when sensor equilibration was achieved, dphi° signals were recorded with FireStingGO2, plotted vs. the O_2_ concentration and fitted with a mathematical model. Phosphorescence lifetime measurements with instruments like Firesting are robust and stable in the measurement geometry and sensor environment [26]. The sensors responded to the partial pressure of O_2_, therefore, the dphi° signal at 0 kPa O_2_ corresponded to vacuum. Sensor response to pO_2_ changes was much greater than signal variability between individual sensors (Figure 2). Therefore, measurement accuracy was high, especially at low O_2_, as is the case with VP.

The following analytical equation (once-off calibration) was obtained, which relates the sample’s O_2_ concentration and the sensor dphi° signals:

O_2_ (%) = 0.034 ∗ dphi°^2^ − 3.413 ∗ dphi° + 85.02 (1)

This equation was used in this study to convert the dphi° readings measured nondestructively in the individual meat packs into the O_2_ (%) values. R^2^ for the fitting was 99.76% (*p* < 0.001). 

### 3.3. Measurement of the Residual O_2_ Content in VP Meat Samples during Chilling Storage

The O_2_ sensors were intended for off-line testing of batches of packaged meat samples which were discarded after testing. In this case, sensor toxicity was not an issue, also considering that high safety and stability and low toxicity had been demonstrated for similar types of sensors [22,23,26].

**Figure 3:**
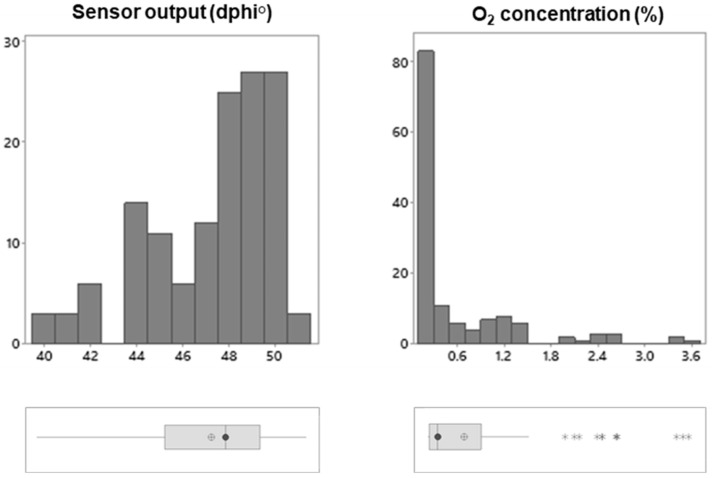
Initial screening of the VP meat samples for the residual O_2_ levels on the day of packaging. Histograms of the data (n = 137) show the distribution of the measured dphi° signals and the calculated O_2_ concentrations (%). The box plots show the interquartile data range, the vertical line and *λ* in the boxes represent the median values and ⊕ represents the mean. The whiskers represent the ranges for the bottom 25% and the top 25% of the data values, excluding the outliers (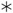).

Initially, sensor signals in all the individual VP meat packs were measured on the day of their packaging at the meat plant (day 1, week 0). The VP meat samples with enclosed sensors were incubated for 20 min in a cold room at 4 °C to equilibrate their temperature, and the sensor signals were measured with FireStingGO2 in a nondestructive contactless manner through the packaging material. The measured dphi° values and the calculated O_2_ content values are presented in Figure 3. The mean sensor dphi° value was 47.24 ± 2.74°, with individual readings ranging from 39.73 to 51.32 and the median of 47.85°. The calculated O_2_ concentration values ranged from 0.12% to 3.57%, with the mean value of 0.60 ± 0.75%. The coefficient of variation for the sensor dphi° signal was 5.8°, for the calculated O_2_ concentration—125.90%. However, the O_2_ data were distributed uniformly around the median value of 0.25%, with 10 samples out of 137 with values in the 1.93–3.57% range.

Consistently with the previous studies [8], the residual O_2_ levels in the majority of the VP meat samples were much lower than 21% but still significantly higher than 0%. This could be due to possible package damage, some air trapped in the packs near the sensor or slow gas equilibration in the VP samples [23]. The optical O_2_ sensing technique can shed light on these issues as it allows VP meat samples to be measured repetitively and over time without affecting their integrity. Time profiles of the O_2_ concentration generated for each sample are much more informative than the conventional once-off destructive testing techniques [23]. 

**Figure 4:**
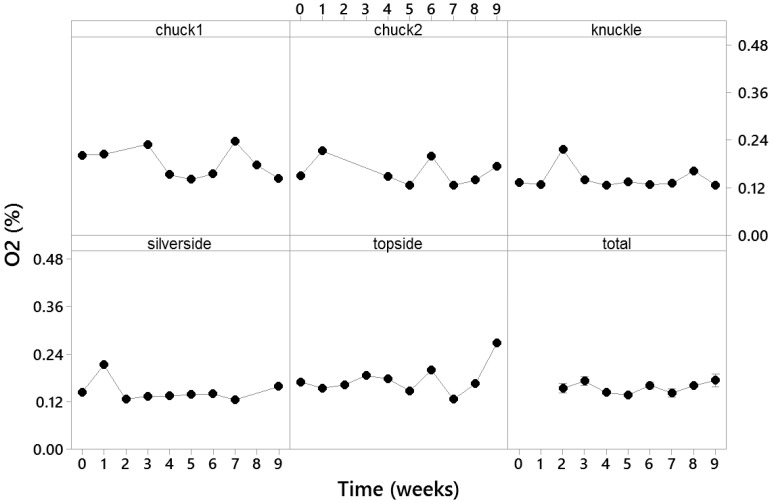
Variation in the residual O_2_ concentration in the VP meat during storage for up to nine weeks at 1 ± 0.5 °C. The data represent the mean value of 15 measurements (three replicates for each of the five samples at each timepoint), and error bars represent thestandard error.

After the initial testing of all the meat packs on day 1, measurements were taken every seven days for a total of nine weeks. Each of the five different types of meat samples was assessed individually for their residual O_2_ content (Figure 4). At each timepoint, the residual O_2_ content was measured nondestructively in the cold room for all the remaining samples. For each VP meat sample, the dphi° signals were read three times with 1-s intervals and then converted into the O_2_ concentrations using the abovementioned calibration equation. 

Overall, the packaged samples did not change significantly in their O_2_ levels during the nine weeks of shelf life compared to week 1 as assessed by the ANOVA analysis (*p*-value ≤ 0.005, α = 0.05). Similarly, differences in the O_2_ concentration between the different meat cuts were not significant. The minimal value measured for the O_2_ concentration was 0.12 ± 0.001% for chuck1 on week 7 and 0.27 ± 0.001% was the maximal value for topside on week 9. The median O_2_ value was 0.13 ± 0.001%. Sensor dphi° signals ranged from 48.28 ± 0.04° (chuck2, week 6) to 51.80 ± 0.006° (topside, week 9), with the median value of 51.80°. It is worth noting that, according to the industry standards, the residual O_2_ levels in commercial VP meat packs should be maintained below 0.3–0.5% [2,3,6]. The results also reflect good stability of sensors in such applications [22,23,26]. 

To demonstrate that the O_2_ sensor system provided meaningful information about the quality of the individual packaged meat samples in a simple and nondestructive manner, we measured several conventional parameters and readouts of meat quality and analysed and compared them to each other. 

### 3.4. Microbiological Analysis during Refrigerated Storage

Raw beef is a highly perishable product; its spoilage due to microbial growth and lipid oxidation during processing and storage are the major factors that cause reduction in the shelf life and foodborne illnesses [27]. Vacuum packaging extends the shelf life of beef eliminating the surrounding air and largely reducing the residual O_2_ levels [28]. Additional benefits include reducing weight loss from evaporation and trimming, preservation of colour, improved hygienic control and palatability due to controlled aging [29]. The dominant bacteria associated with spoilage in VP and cold-stored beef are lactic acid bacteria (LAB), Enterobacteriaceae and Brochothrix thermosphacta. Psychrophilic and psychrotrophic species growth produces gas at the temperatures of 1–2 °C, causing the swelling of vacuum packages after 14 days [30].

**Figure 5:**
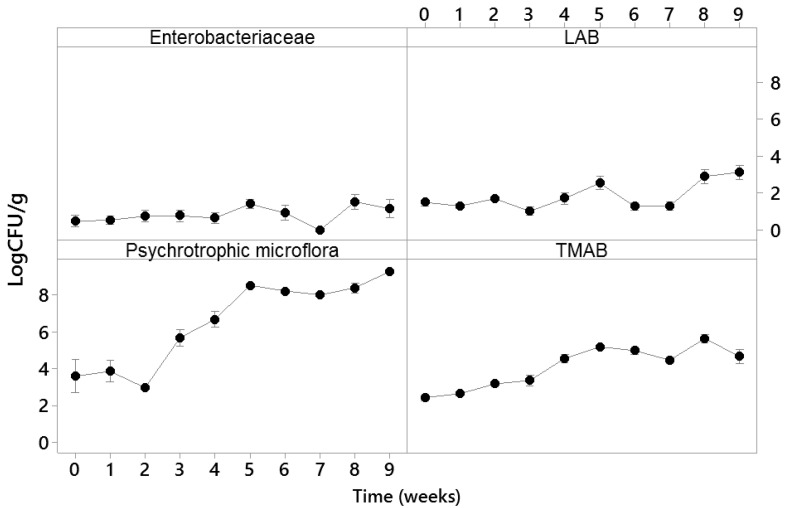
Changes in the microflora in the different VP meats over the nine weeks of their storage. The datapoints represent the mean values for three replicates, the bars represent the standard errors. LAB: lactic acid bacteria; TMAB: total mesophilic aerobic bacteria.

The development of meat microbiota during the shelf life storage of our samples was followed by destructive weekly sampling of 10 g from one of each of the five types of VP meats for each bacterial population assayed over nine weeks. The data of the microbiological analysis during the shelf life of VP meat (Figure 5) showed a steady increase in LAB, TMAB and psychrotrophic bacteria. Typically, psychrotrophic bacteria initially dominate the microbiota of VP meat, with the initial value of 3.59 ± 2.83 log CFU/g after packaging to 5.08 ± 0.91 log CFU/g at the end of nine weeks of storage. The TMAB changed from 2.72 ± 0.23 log CFU/g on the packing day (day 1, week 0) to 5.09 ± 0.91 log CFU/g on week 9. The population of LAB was generally low, ranging from 1.02 ± 0.69 to 2.54 ± 1.12 log CFU/g in the first seven weeks and reaching 3.13 ± 1.2 log CFU/g nine weeks after packaging. 

**Figure 6:**
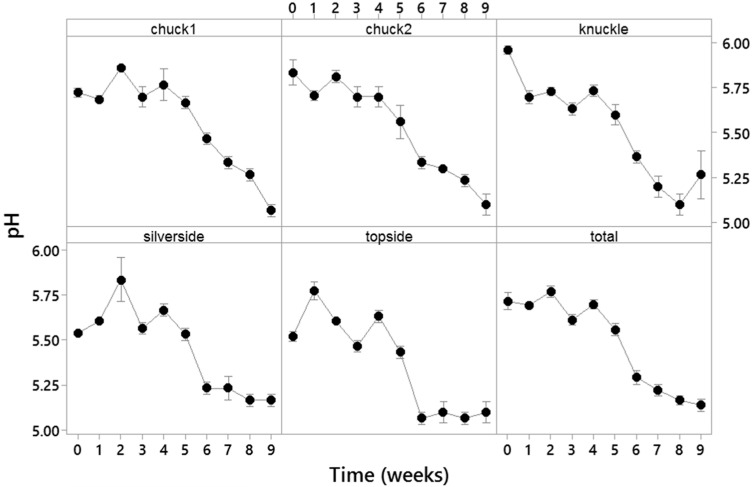
Changes in pH in the VP meats over the nine weeks of their storage. The datapoints represent the means for three replicates. The bars represent the standard error of the means.

The microbiota developing in meat during the shelf life can cause the variation of its pH values [31]. For VP meats, decreases in pH values may be due to low O_2_ levels favouring the growth of facultative anaerobic bacteria such as LAB [32]. In this study, the meat samples’ pH values of around 5.72 ± 0.18 at the start of the storage decreased to 5.14 ± 0.13 after nine weeks of storage. Such decreases in meat pH values were observed for all the meat cuts assayed (Figure 6).

### 3.5. Colorimetric Analysis

**Figure 7:**
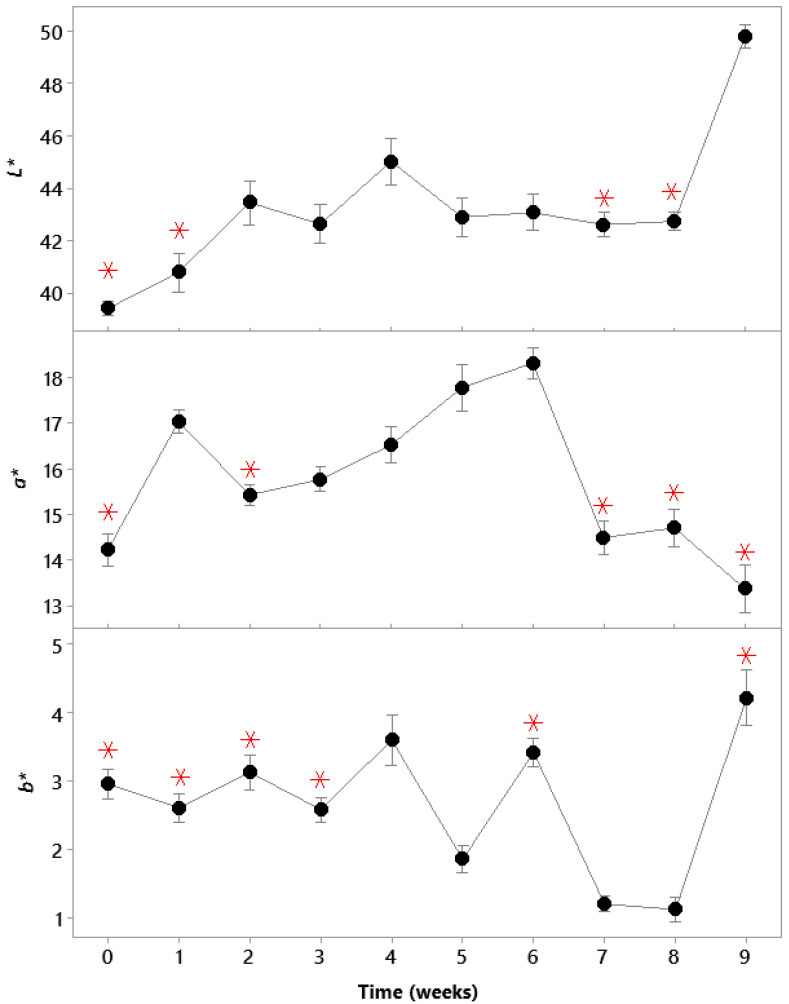
Changes in the CIE L*, a* and b* values of the VP meat during nine weeks of storage. The means labelled with the red asterisk symbols (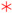) were not significantly different from the control level mean at time 0 (day 1, week 1) according to the ANOVA test (Dunnet comparisons, α = 0.05). The error bars represent the standard errors of the means.

Changes in the surface L*, a* and b* (lightness, redness and yellowness, respectively) values of the samples during storage are shown in Figure 7. The CIE L* values increased significantly (*p* < 0.001) during storage, changing from 39.43 ± 1.46 on the packing day to 49.81 ± 1.40 on week 9, thus indicating the meat samples were lighter at the end of storage. The a* values of the samples decreased from 14.23 ± 1.97 on week 1 to 13.36 ± 1.65 on week 9, with a significant increase (*p* < 0.001) in weeks 3–6 and a peak of 18.32 ± 2.39 on week 6. The decrease in a* may have been due to oxidation of myoglobin in VP meat [33], while water loss by meat might have also led to the accumulation of myoglobin at the surface [34]. The b* values of the samples changed from 2.96 ± 1.19 on the packing day to 4.22 ± 1.26 on week 9, with the minimal value of 1.13 ± 0.55 on week 8 and the maximal value of 22 ± 1.26 at the end of the storage period. The variations in b* over the storage period could have been related to the intensity of the oxidation process that takes place during storage, which may increase the yellowness of samples via rancidity [33]. 

## 4. Conclusions

Elevated O_2_ concentration in packaged foods can facilitate the growth of aerobic bacteria and oxidative reactions which induce the development of off-odours, undesirable colour, deterioration and reduction of the nutritional value of the product. Sensors for nondestructive monitoring of the residual O_2_ in packaged meat products are useful for assessing their quality and process optimization [17]. Sensors can easily be employed in the meat industry for nondestructive, fast, quantitative and real-time monitoring of the residual O_2_ levels in packaged products [2,8,17]. In this research, we modified the previous version of this method, which used commercial sensor stickers [8], by producing low-cost in-house-made disposable sensor dots in large quantities. The production of such O_2_ sensors is facile, easy and reproducible. The manufactured sensor dots were used on the disposable basis in an industrial trial conducted at an industrial meat processing plant. The sensor dots were applied directly on meat cuts prior to their packaging on an industrial vacuum packaging machine.

The sensor dots produced allowed for nondestructive and repetitive measurements of the residual O_2_ levels in VP fresh meat products using an inexpensive autonomous handheld reader, FireStingGO2. The measurements took just a few seconds per sample, and they were performed on-site, in a cold room without altering the temperature and storage conditions of the food samples. Several other established destructive quality tests for VP meat samples were also carried out and showed the anticipated results and time profiles.

## Data Availability

The data supporting the reported results can be obtained from the corresponding author upon reasonable request.
